# The complete chloroplast genome of *Crepis rigescens* (Cichorioideae), a traditional Chinese medicinal plant

**DOI:** 10.1080/23802359.2022.2068978

**Published:** 2022-05-02

**Authors:** Peng Li, Xiaoai Fang, Xiaoqing Liang

**Affiliations:** Department of Pharmacy, School of Medicine, Xi’an International University, Xi’an, China

**Keywords:** *Crepis rigescens*, Cichorioideae, chloroplast genome, Illumina sequencing, BI phylogenetic analysis

## Abstract

*Crepis rigescens*, Diels 1921 is a traditional Chinese medicinal plant of Cichorioideae, which contains many chemicals, such as friedelin, *β*-sitosterol, stigmasterol, chlorogenic acid, and flavonoids, and so on, which has the characteristics of high medicinal value and small side effect. *Crepis rigescens* was used as folk medicines for anti-bacterial, and anti-oxidation, which also had a potential curative effect in preventing cardiovascular disease and anti-tumor. Illumina paired-end reads data were used to assemble the complete chloroplast (cp) genome. 14,425,796 raw paired-end reads and the length distribution in 124,685 bp, including a large single copy (LSC) region of 82,924 bp, a small single copy (SSC) region of 18,150 bp, and a pair of inverted repeat (IRs) regions of 25,128 bp. Besides, 10 protein-coding genes (PCGs) genes and 6 tRNAs genes possess a single intron, while *clpP* and *ycf3* have a couple of introns. Based on the concatenated coding sequences of cp PCGs, the phylogenetic analysis showed that *C. rigescens* and *Hypochaeris radicata* (MH746729) are closely related to each other within the family Cichorioideae.

*Crepis rigescens* is a traditional Chinese medicinal plant of Cichorioideae, which contains many chemicals, such as friedelin, *β*-sitosterol, stigmasterol, Chlorogenic acid, and flavonoids, and so on, which have the characteristics of high medicinal value and small side effect (Kisiel et al. 2000; Bo et al. 2012; Ma et al. 2015). *Crepis rigescens* was used as folk medicines for anti-bacterial, and anti-oxidation, which also had a potential curative effect in preventing cardiovascular disease and anti-tumor (Bo et al., 2012; Tsoukalas et al. 2014).

The complete chloroplast (cp) genome consists of a pair of inverted repeats (IRs), separated by a large single-copy region (LSC) and a small single-copy region (SSC), these four parts constitute a conserved structure of the complete cp genome (Wolfe et al. 1992; Lee et al. 2007). This report will be very important for studying the phylogenetic relationships between *C. rigescens* and Cichorioideae.

The fresh leaves of *C. rigescens* were collected at Bijie, Guizhou, China (27.31 N, 105.23 E) (This plant is a common plant, and we do artificial cultivation in plants. Hence, ethical approval is not required.) and were preserved in 95% ethanol, and then transferred to a laboratory at −20 °C for long-term storage at Xi’an International University (https://www.xaiu.edu.cn/, specimen voucher: HYS 210501, Xiaoqing Liang, xiaoqing870406@163.com). The genomic DNA was extracted with the modified CTAB method (Doyle and Doyle 1987). The complete Chloroplast genome sequencing and assembly were performed by Shaanxi Airui Biological Technology Co., Ltd. (Shaanxi, China). Total genomic DNA was isolated from approximately 100 mg of fresh leaves of *C. rigescens* using the DNeasy Plant MiniKit (Qiagen, CA, USA). After the detection of DNA purity and integrity, high-quality DNA was used for library construction and sequenced using Illumina Noveseq with a paired-end 150 strategy. Genomic DNA was used for sequencing with Illumina HiSeq X Ten Sequencing System (Illumina, CA, USA). The raw sequencing data were quality-trimmed with Geneious R11 (Biomatter Ltd., Auckland, New Zealand) and conducted with the program MITObim v 1.9 (https://github.com/chrishah/MITObim) (Hahn et al. 2013). The chloroplast genome of *Crepidiastrum lanceolatum* (MK358413) was used as the initial reference. While 4 Asteroideae were selected as outgroups (*Artemisia scoparia* MN385624, *Galinsoga parviflora* MK737938, *Leucanthemum vulgare* MN989913, and *DTithonia diversifolia* MT576958). After the assembly, the trimmed raw data were mapped to the assembled chloroplast sequence to check the assembly quality and coverage.

We assembled a 124,685 bp (GC content accounts for 37.8%) circular chloroplast genome from 14,425,796 raw paired-end reads. In addition, the length of LSC region, SSC region and IR regions distribution in 82,924 bp (GC, 36.0%), 18150 bp (GC, 31.6%), and 25,128 bp (GC, 43.0%), respectively. Based on the web-based tool OGDRaw v1.2 (https://chlorobox.mpimp-golm.mpg.de/OGDraw.html) to generate the complete cp genome (Lohse et al. 2013).

The genome sequence data that support the findings of this study are openly available in GenBank of NCBI at [https://www.ncbi.nlm.nih.gov] (https://www.ncbi.nlm.nih.gov/) under the accession No. OM320794. The associated BioProject, SRA, and Bio-Sample numbers are PRJNA786641, SRP349367, and SAMN23673929, respectively.

From the sequencing result, we obtained 114 complete genes, including 80 PCGs (protein-coding genes), 30 tRNAs (transfer RNAs) genes, and 4 rRNAs (ribosomal RNAs) genes. In total, 10 PCG genes (*atpF*, *ndhA*, *ndhB, petB*, *petD*, *rpl16, rpoC1*, *rps12*, and *rps16*) harbor a single intron. 68 PCG genes have no intron. 6 tRNAs genes (*trnA-UGC*, *trnG-UCC*, *trnI-GAU*, *trnK-UUU*, *trnL-UAA*, and *trnV-UAC*) harbor a single intron, and *ClpP* and *ycf3* harbor two introns.

Based on the concatenated 15 cp PCGs from 36 published species of Cichorioideae and 4 Asteroideae were selected as outgroups. We constructed a Bayesian Inference (BI) phylogenetic tree (Figure 1) using MrBayes v3.1.2 (Milne et al. 2009) program integrated with TOPALi V2.5 software (Ronquist and Huelsenbeck 2003) to further study the phylogenetic position of *C. rigescens.* From the BI phylogenetic tree analysis, we find that *C. rigescens* and *Hypochaeris radicata* (MH746729) are closely related to each other within the family Cichorioideae (Figure 1).

**Figure 1.**
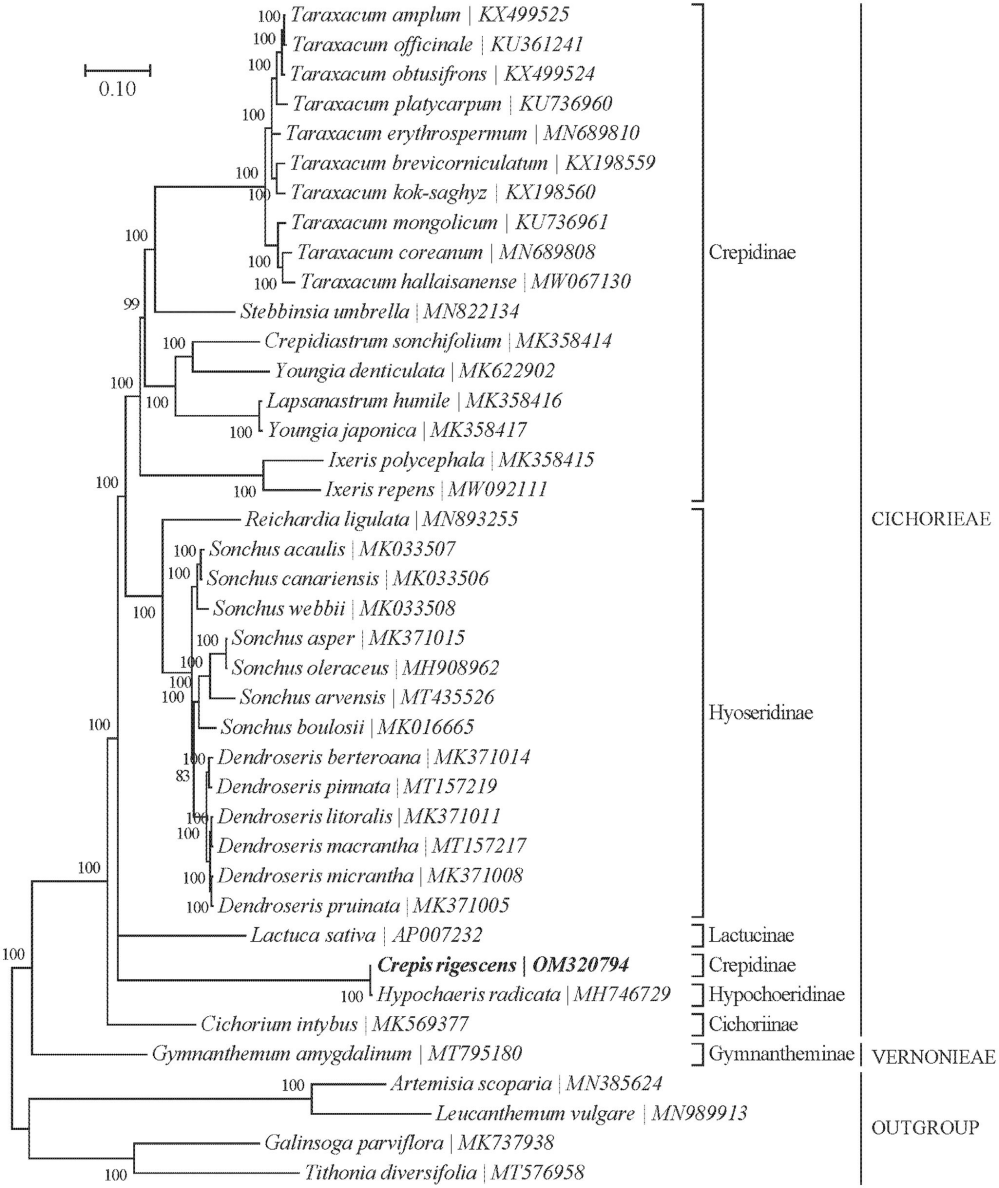
Phylogenetic position of *Crepis rigescens* based on a comparison with the complete mitochondrial genome sequences of 36 other Cichorioideae species and 4 Asteroideae as outgroups. The analysis was performed using MrBayes v3.1.2 program integrated with TOPALi V2.5 software. The accession number for each species is indicated after the scientific name.

## Authors’ contributions

Peng Li’s substantial contributions to the conception or design of the work. Xiaoai Fang’s contributions are cultivation management and collection of plants. Xiaoqing Liang’s contribution is the interpretation of data for the work.

## Data Availability

The data that support the findings of this study are openly available in the Genbank database at https://www.ncbi.nlm.nih.gov/under the accession number OM320794. The associated BioProject, SRA, and BioSample numbers are PRJNA786641, SRP349367, and SAMN23673929 respectively.
